# Fluoride application in middle childhood. A cross-sectional study

**DOI:** 10.1007/s00784-025-06477-0

**Published:** 2025-08-07

**Authors:** Vera Wiesmüller, Stephanie Müller, Amelie Großhans, Ulrike Lepperdinger, Ines Kapferer-Seebacher

**Affiliations:** 1 University Hospital for Conservative Dentistry and Periodontology, Medical University of Innsbruck, Anichstr. 35, Innsbruck, A- 6020 Austria; 2 Hall in Tyrol, Austria

**Keywords:** Dental caries, Prevention, Fluorides, Children, Dentistry for children, Toothpaste

## Abstract

**Objectives:**

Fluoride application is crucial for caries prevention; yet guidelines for middle childhood remain limited. This study evaluated the daily fluoride exposure of children aged six to 12 years and compare the findings with the available recommendations.

**Methods:**

Children applied their usual amount of toothpaste to a manual toothbrush, which was weighed before and after application. Additionally, data on residence in relation to fluoridation of tap water, frequency of oral hygiene practices, the oral hygiene products used, additional fluoride supplementation, and parental knowledge regarding fluoride prophylaxis was collected to determine daily fluoride exposure. The data were analysed in accordance with current recommendations.

**Results:**

The study included 458 children aged 8.0 ± 1.77 years. Age-appropriate toothpaste was used by 76.4%. The mean quantity of toothpaste utilised was 0.42 ± 0.27, while 0.5–0.75 g are recommended. The mean daily fluoride intake via toothpaste was found to be 1.01 ± 0.81 mg. 94.1% of the study cohort does not meet the recommendations of the German Society for Preventive Dentistry. Only a third of the cohort used at least one supplementary fluoride source in addition to toothpaste (37.7%). 43.48% of legal guardians expressed the opinion that fluoride prophylaxis is recommended for their child.

**Conclusions:**

The results highlight an urgent need for parental education.

**Clinical relevance:**

In an area of low-fluoridated drinking water children over six years should use a full brush length (>0.5 g) of fluoridated toothpaste (approximately 1450 ppm) twice daily, along with an additional fluoride source such as fluoridated salt, mouthwashes or gels. Study register of the University Hospital Innsbruck (clinical trial registration number 20220331-2872)

## Introduction

In 2019, dental caries was the most prevalent disease among children aged 0 to 14 years, with an estimated global burden of 0.5 billion cases (ranging from 0.4 to 1.6 billion) [1]. Fluoride application remains a cornerstone of caries prevention strategies [2]. Although systemic fluoridation played a pivotal role in the early phases of fluoride-based prophylaxis, the efficacy of daily topical fluoride application is now well established and is recommended for all ages. Topically applied fluoride is incorporated into the dental enamel through the dynamic processes of demineralization and remineralization, resulting in the formation of fluorapatite—a crystalline structure more resistant to acid dissolution than hydroxyapatite, particularly in the acidic environment generated by the metabolism of dietary sugars by oral bacteria. Fluoride can be applied at home via toothpaste, mouth rinses, or gels, while professional in-office treatments often involve the use of fluoride varnishes. In regions with limited access to naturally fluoridated sources, community-based fluoridation programs—utilizing fluoridated water, salt, or milk—may be implemented as effective public health measures [3, 4]. For instance, fluoridated table salt has been shown to elevate fluoride concentrations in saliva and dental plaque for approximately one hour post-consumption, thereby offering localized caries protection comparable to that of fluoridated toothpaste [5].

Middle childhood (ages six to 12) is often called the ‘forgotten years’ because most research focuses on early childhood development or adolescent growth [6, 7]. A recent recommendation from the German Nationwide Network “Healthy into Life” for fluoride prophylaxis, which was immediately implemented by the dental industry, focused on infancy and early childhood but omitted guidance for children over six [8]. In 2024, the German Society for Preventive Dentistry closed this information gap, recommending children aged six to 12 to use a 1 to 1.5 cm strand of toothpaste (0.5–0.75 g) with at least 1400 ppm administered twice daily. Middle-aged children with an elevated caries risk should additionally utilise a mouth rinse with ≥ 250 ppm fluoride on a daily or twice-daily basis or a 1.25% fluoride gel weekly. Fluoridated table salt is recommended in all age groups after the eruption of the first primary tooth [9].

The European Food Safety Authority (EFSA) recommends a daily intake of 0.05 mg per kilogram of body weight for all ages [10]. Despite fluoride’s caries-protective effect being only attributable to its topical application, and its lack of an essential biological function, the EFSA has chosen to base its recommendation on the daily fluoride quantity per kilogram of body weight, encompassing all dietary and non-dietary sources of fluoride.

Critics of fluoride use warn against fluorosis, a hypomineralization of enamel causing discolouration, ranging from - in most cases - mild white lines or patches in the enamel to severe mottling possibly involving brown staining. Enamel fluorosis of the aesthetically important anterior teeth is caused by excessive fluoride intake during the first five years of life, with the most critical period being the first three years [11, 12]. In the context of fluorosis prophylaxis, it is essential to consider the fluoride content of drinking water, given the considerable variation observed across different regions. Fluoridation of tap water represents a cost-effective prevention strategy, with an optimal concentration for this purpose is 0.7-1.0 mg/L [11]. The majority of European countries have favoured alternative caries-protective measures, resulting in varying fluoride levels [11]. Recent studies have disproven another criticism of fluorides, as no evidence has been found indicating an effect of early fluoride exposure on children’s cognitive development [13].

Using the right fluoride dosage is a challenge for parents and caregivers, especially in the case of middle-aged children, who lack standardised dosage recommendations. This study aimed to evaluate the daily fluoride exposure of children aged six to 12 years, compare the results to existing recommendations and assess parental knowledge regarding fluoride prophylaxis.

## Methods

This study was approved by the Ethics committee of the Medical University of Innsbruck, Austria (EK 1417/2021) and was registered at the study register of the University Hospital Innsbruck (clinical trial registration number 20220331-2872). The study was conducted in accordance with the 1964 Helsinki declaration and its later amendments. Legal guardians provided written informed consent prior to inclusion.

Children aged six to twelve years, along with their parents or both—depending on who was responsible for toothpaste application at home—were recruited from public locations in Innsbruck, Austria (e.g., shopping centers and primary schools). To minimize selection bias, recruitment was conducted across multiple city districts to ensure inclusion of participants from diverse socioeconomic backgrounds. All children within the specified age range were eligible to participate; no exclusion criteria were applied. Participants were instructed to dispense the typical amount of toothpaste they would use at home onto an age-appropriate manual toothbrush. Various commercially available toothpastes were used in this study, some of which were not age-specific (Elmex© Baby/Junior, CP GABA, Hamburg, Germany; GUM Junior 6+, Sunstar©, Etoy, Switzerland), with the common criterion being similar dispensing orifice size and absence of built-in portion control. Only manual toothbrushes of comparable size were utilized (Kids Soft, TePe©, Malmö, Sweden; Junior, Oral-B©, Procter & Gamble, Schwalbach, Germany; Junior 6+, Sunstar©, Etoy, Switzerland). After application, participants were asked whether the dispensed amount visually corresponded to their usual application at home. Repetition was permitted until participants were satisfied with the result. Each toothbrush was weighed before and after toothpaste application using a precision scale (readability d = 0.01 g; Sartorius Extend ED4202S-0CE©, Sartorius AG, Göttingen, Germany).

Additionally, a structured interview with the parents was conducted to collect data necessary for calculating the child’s daily fluoride intake. This included information on place of residence (to assess tap water fluoridation), body weight, oral hygiene frequency, oral hygiene products used at home, and any supplemental fluoride measures. To ensure reliable recall of oral hygiene products, visual boards displaying images of all regionally available toothpastes were presented. Fluoride content in local tap water was obtained from relevant authorities or via the official drinking water information portal (https://www.trinkwasserinfo.at). Finally, parents were asked whether they believed fluoride prophylaxis was appropriate for their child and what they considered an appropriate fluoride dosage.

According to the recommendations of the German Nutrition Society, drinking quantities of 940 ml/day were assumed for six-year-olds, 970 ml/day for seven- to nine-year-olds and 1170 ml/day for ten- to 12-year-olds [14]. For calculating the daily fluoride intake via drinking water the fluoride content of the drinking water in the place of residence was obtained from the competent authority. For the calculation of fluoride intake from oral hygiene products or systemic fluoride tablets, the exact brand names and dosages were documented. If fluoridated table salt was used, a daily fluoride intake was calculated according to the WHO recommended dose of 2 g/day of table salt with 250 mg/kg fluoride, which corresponds to an amount of 0.5 mg per day [15].This allowed the calculation of daily fluoride exposure of participants.

Furthermore, it was analysed to what extent the toothpaste and fluoride amounts used are in line with the existing recommendations of (I) the German Society of Preventive Dentistry, which stipulates the use of 0.5 to 0.75 g toothpaste with a fluoride concentration of at least 1400 ppm twice daily in combination with the use of fluoridated table salt and (II) the EFSA recommendation for a daily use of 0.05 mg fluoride per kilogram body weight, including intake from dietary and use of non-dietary sources such as topical application during daily oral hygiene.

Data was collected from April to August 2022. Microsoft^®^ Excel (Microsoft Corp. Version 16.77.1, Redmond, Washington, USA) was used for data collection, IBM SPSS Statistics (IBM Corp. IBM SPSS Statistics for Windows, Version 29.0, Armonk, New York, USA) was used for calculations and for graph creation. Only fully completed examinations were included in the analysis, with the exception of body weight data. For descriptive analyses metric variables were presented as mean and standard deviations. Kruskal-Wallis Test, Chi^2^ Tests and Odds-Ratio were employed to conduct group comparisons. To assess the association between age and toothpaste amount, Spearman’s rank correlation coefficient (ρ) and a simple linear regression analysis was performed.

## Results

The study included 458 children, aged 8.1 ± 1.76 years, of which 220 (48.0%) were female and 238 (52.0%) were male. For 107 children, reliable body weight data were unavailable at the time of the study; therefore, fluoride intake per kilogram of body weight was not calculated for these participants. The majority of children who participated in the study were from Tyrol (Austria), while smaller proportions came from Italy (4.1%) and other Austrian provinces (1.1%). It is noteworthy that these regions did not implement regulated drinking water fluoridation and exhibited low fluoride levels. The average fluoride level of tap water in the participants’ places of residence was 0.19 ± 0.17 mg/l (range 0.01–1.0 mg/l).

**Fig. 1:**
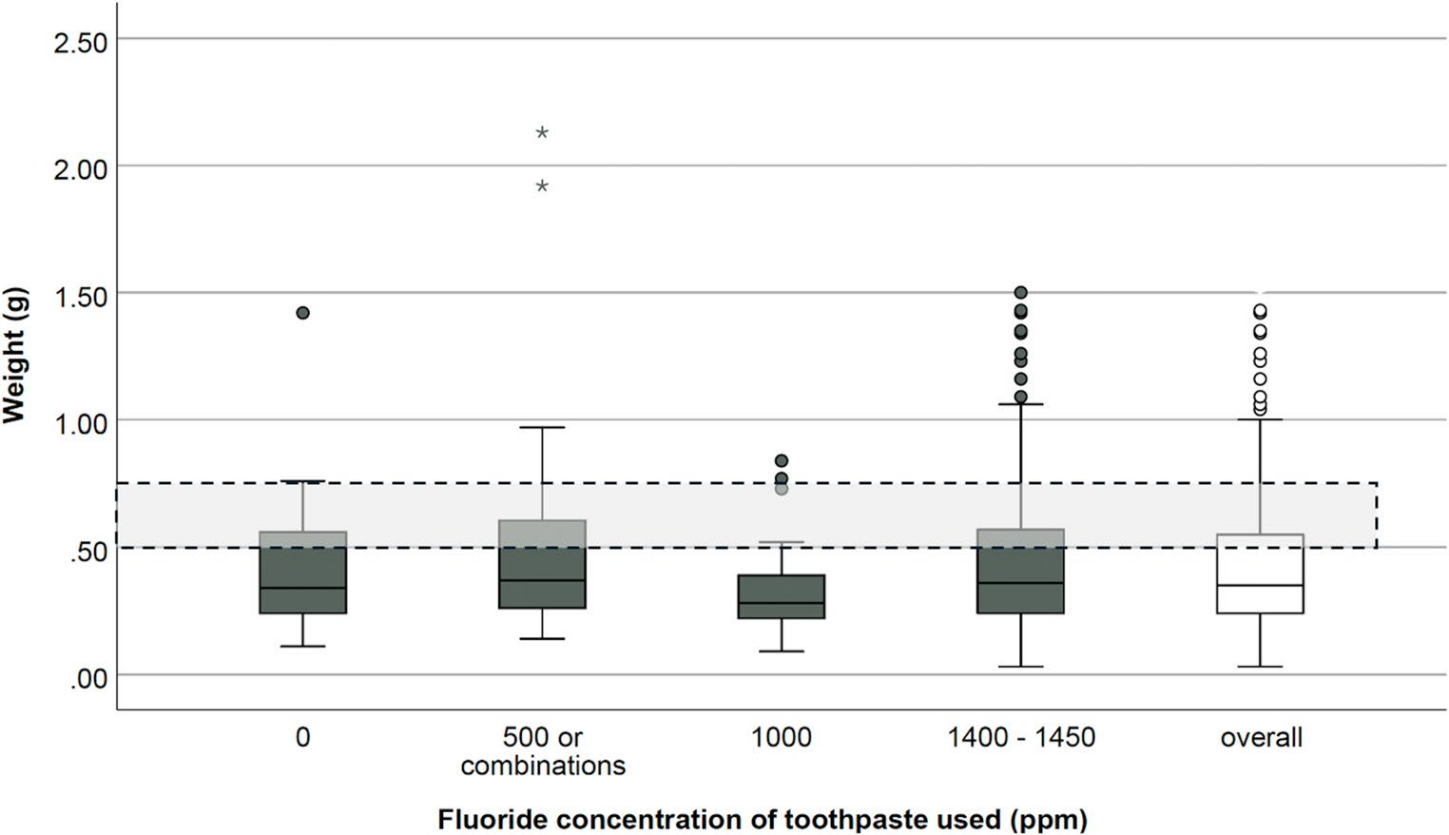
Weight of toothpaste used per application with different fluoride concentrations. The results demonstrate that there was no adjustment in the amount of toothpaste used based on different fluoride concentrations (*p* > 0.05). The majority of children (70.6%) used less toothpaste than recommended by the German Society of Preventive Dentistry which stipulates the use of 0.5 to 0.75 g toothpaste. Only 23.1% met the recommended fluoride exposure via toothpaste

On average, 0.42 ± 0.27 g of toothpaste, a minimum of 0.03 g and a maximum of 2.13 g, was used. A fluoride-containing toothpaste with 1400–1450 ppm was used by 76.4% (350) of participants, 9.6% using 1000 ppm toothpaste. A total of 10.3% of participants used a lower-dose fluoridated toothpaste (500 ppm) or an alternating combination of fluoridated and non-fluoridated toothpaste. In contrast, 3.7% of participating children exclusively used a non-fluoridated toothpaste. There was no statistically significant difference in the amount of toothpaste used between the groups with varying fluoride concentrations (*p* = 0.209) (Fig. 1). A weak but statistically significant positive association was observed between age and the amount of toothpaste (ρ = 0.17; *p* < 0.001). Linear regression analysis confirmed this relationship, although the explained variance was low (R² = 0.009; *p* = 0.040), suggesting that age accounts for less than 1% of the variability in the amount of toothpaste used.

Thus, an average of 1.01 ± 0.81 mg fluoride (ranging from 0.00 to 6.22 mg) was applied daily via the carrier medium toothpaste. The overall daily intake of fluoride from oral care products, drinking water and fluoridated foods (such as salt) was calculated to be 0.03–8.08 mg/day, with an average of 1.54 ± 1.03 mg. While 62.7% of the participants used only toothpaste for fluoride prophylaxis, 30.3% used one additional fluoride source and 7% used two or more sources in addition to toothpaste for fluoride prophylaxis. The most common additional source of fluoride was fluoridated table salt, accounting for 27.1% of the sample. Other fluoride-sources were largely avoided: 15.3% of the participants reported using fluoridated mouthwash, 0.4% used fluoride tablets, and 2.2% reported other sources of fluoride, such as weekly gel application.

Out of the 458 guardians surveyed, only 200 (43.7%) stated that, according to their level of knowledge, fluoride prophylaxis is recommended for their child, 201 (43.9%) did not have an opinion on this topic, and 57 (12.5%) stated that, in their opinion, fluoride prophylaxis is not recommended for their child. When queried further on their awareness regarding the fluoride content of the toothpaste utilized, 41.3% of parents indicated a lack of knowledge in this regard. Of the 270 parents who indicated that they were aware of the fluoride content of the toothpaste used, only 186 (40.6% of the total cohort) were correct in their assumption. 30.7% of the 270 respondents incorrectly believed that the toothpaste was fluoride-free, yet it actually contained fluoride. Conversely, only one individual identified that their child was using a fluoridated toothpaste that was actually fluoride-free. Summarising, only 40.6% of guardians were correctly informed about the fluoride content of the toothpaste currently used by their children.

### Accordance with the recommendation of the German society for preventive dentistry

**Fig. 2:**
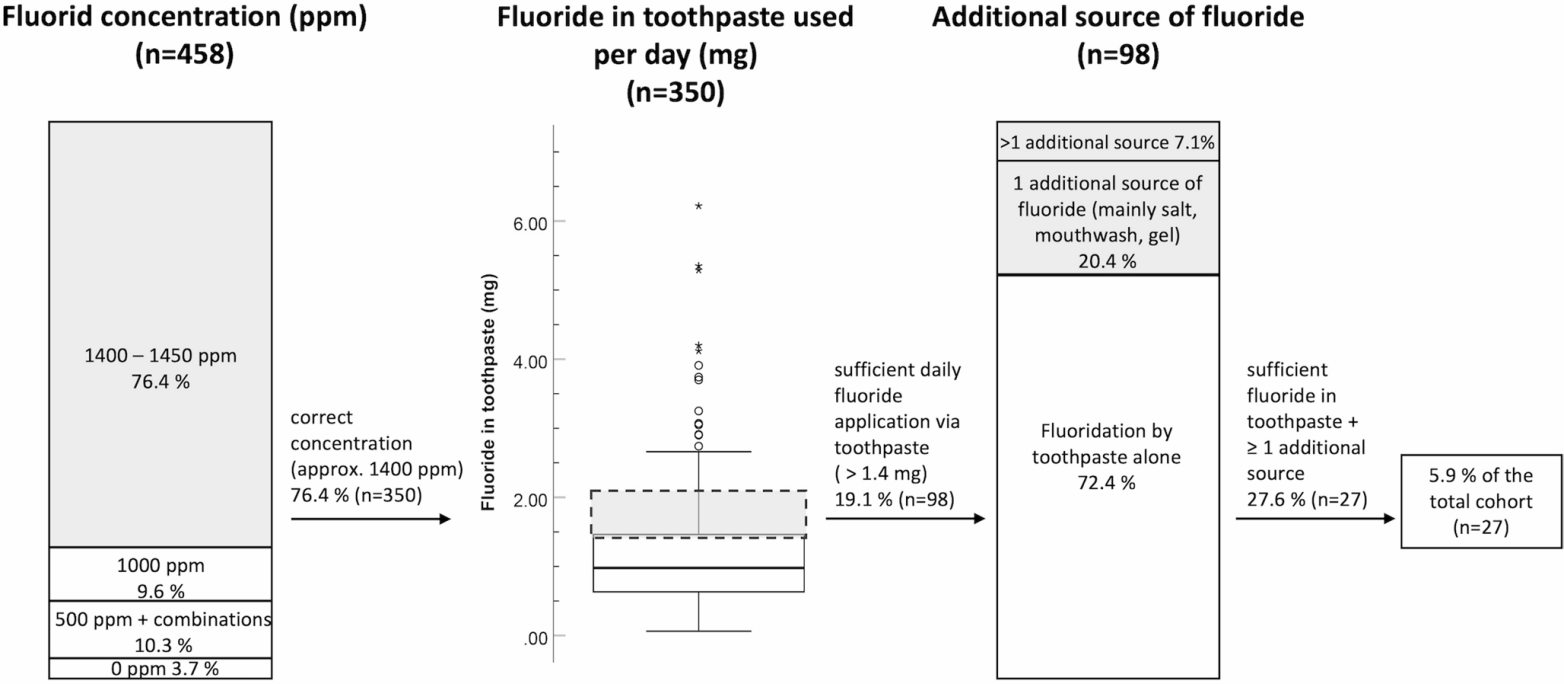
Accordance with the recommendation of the German Society for Preventive Dentistry. The German Society for Preventive Dentistry recommends for middle-aged children the use of 0.5 to 0.75 g toothpaste with a fluoride concentration of at least 1400 ppm two times per day in combination with the use of fluoridated table salt (recommendations highlighted in grey). Only 5.9% of our cohort met all recommended criteria

The study’s findings indicate that 94.1% of the study cohort does not meet the specified criteria of the recommendation of the German Society for Preventive Dentistry (Fig. 2). Only 18.3% (*n* = 84) used the adequate amount of toothpaste for caries prevention, while 11.1% (*n* = 51) used a greater quantity than recommended. In accordance with the aforementioned guideline, a daily application of fluoride via toothpaste of 1.4 to 2.1 mg is recommended. Our findings indicate that the mean daily fluoride exposure via toothpaste is 1.01 ± 0.81 mg, with 86.9% of subjects not achieving the recommended daily amount of fluoride via toothpaste for caries prevention (Fig. 2).

The recommendation entails the utilization of fluoridated table salt as an additional source of fluoridation. This was employed by 27.1% of the study cohort. In addition to toothpaste, 30.3% of participants utilized one alternative fluoride source, including salt, mouthwashes, fluoride gels or tablets. A further 7% used two or more supplementary fluoride carriers. The proportion of participants who consume a sufficient amount of fluoride from toothpaste and use an additional fluoride source in accordance with the recommended regimen is 5.9% of the total cohort (Fig. 2).

### Accordance with the recommendation of the European food safety authority

Based on complete data information including body weight the daily fluoride intake of 351 children was analysed. An average of 0.06 ± 0.04 of fluoride per kilogramme of body weight was consumed daily from drinking water, oral care products and additional fluoridated products. The results showed that only 47.0% (165/351) of the children met or exceeded the recommended daily fluoride intake of 0.05 mg/kg.

### Decisive factors for an adequate supply of fluorides

In the cohort under investigation (*n* = 458), 350 children used an age-appropriate toothpaste with a fluoride concentration of approximately 1400 ppm. A total of 28.0% of this group consumed the amount of fluoride via the toothpaste recommended by the German Society for Preventive Dentistry. Among the 108 children who used a toothpaste with a lower fluoride concentration, 7.4% achieved the desired amount of fluoride through toothpaste, indicating an elevated probability of underdosing with a toothpaste that is not age-appropriate (OR = 4.86, 95% CI [2.28, 10.36]; *p* < 0.001).

In the analysis of cases with complete body weight information (*n* = 351), it was demonstrated that the subgroup of children who used at least the recommended amount of toothpaste (0.5–0.75 g) (*n* = 92) achieved the EFSA target value for effective caries prophylaxis in 77.2% of cases. A decisive factor was, again, the fluoride concentration of the toothpaste, which differed statistically significantly between this subgroup and the remaining 22.8% (*p* = 0.007). A comparison of the drinking water fluoride content between these two groups revealed no statistically significant difference (*p* = 0.549).

**Table 1:** Daily fluoride consumption (mg/kg body weight)

		Amount of toothpaste used		
	Total (*n* = 351)	< 0.25 g (*n* = 107)	0.25–0.5 g (“pea-size”) (*n* = 152)	> 0.5 g (*n* = 92)
Daily fluoride, *mg/kg*	0.055 ± 0.040	0.037 ± 0.022	0.053 ± 0.031	0.080 ± 0.054
Recommended dosage achieved or exceeded, *n (%)*	165 (47.0)	24 (22.4)	70 (46.1)	71 (77.2)
Propability of being under-dosed, *Odds*	1.12	3.46	1.17	0.30

The European Food Safety Authority recommends a daily fluoride intake of 0.05 mg/kg body weight from all sources, including dietary intake and topical application. In this study, 47.0% of children met this recommendation. Children not using an appropriate amount of toothpaste have an increased probability of being under-dosed

As demonstrated in Table 1, children who used less than a pea-sized portion (0.25 g) of toothpaste had an increased probability of being under-dosed, not reaching the ideal amount of daily fluoride for caries prevention (Odds 3.46). Participants who used between 0.25 and 0.5 g of toothpaste were more likely to be under-dosed (Odds = 1.17), while the use of toothpaste in quantities greater than 0.5 g was associated with a reduced likelihood of underdosing according to the EFSA guidelines (Odds = 0.30). In summary, the utilisation of a quantity of fluoride that is less than the recommended 0.5 g has been found to be associated with a higher chance of underdosing with respect to the recommended daily amount of fluoride for caries prevention (OR = 6.31, 95% CI [3.58, 11.13]; *p* < 0.001).

## Discussion

Middle childhood (ages six to twelve) is often described as the “forgotten years” in research, as developmental and psychological studies focus on early childhood or adolescence [6, 7]. The objectives of the present study were therefore to evaluate the fluoride exposure in middle-aged children, compare it with existing recommendations, and assess parental knowledge regarding fluoride prophylaxis. The findings indicate that (I) the use of supplementary fluoride sources beyond toothpaste is minimal, (II) parental awareness of fluoride prophylaxis is insufficient, and (III) the daily amount of fluoride administered is too low for effective caries prevention in the majority of the cohort.

A total of 99.8% of the study population resided in areas with low fluoride concentrations in drinking water, with a mean fluoride level of 0.19 ± 0.17 mg/L—well below the recommended 1–1.5 mg/L for caries prevention [16–18]. In such regions, fluoridated toothpaste serves as the primary fluoride source. A study in England found that, in low-fluoride areas, toothpaste accounted for up to 93% of fluoride intake [19]. Similarly, in the present cohort, toothpaste was the dominant fluoride source, with 30.7% of children using one additional fluoride source and 7% using two.

Fluoridated table salt use was low (27.1%), in contrast to neighbouring countries where it plays a key role in caries prevention. In Switzerland, the use of 250 mg fluoride/kg table salt led to a 71–78% caries reduction (1974–1987), prompting a nationwide recommendation [20]. As of 2015, the market share of fluoridated and iodized salt in Switzerland exceeded 89%, whereas in Germany, fluoridated salt accounted for 63% of sales in 2004 [21]. The acceptance of fluoridated table salt in Austria remains low in comparison. An enquiry at Salinen Austria AG, Ebensee revealed that the portfolio share of table salt with fluoride is only approximately 3.8% of the quantity, indicating untapped potential for caries prevention.

A preference for the use of other fluoride sources such as mouthwashes instead of table salt was not found in this study. Only 15.3% of the children used mouth rinses. A recent systematic review concluded that supervised rinse programmes result in an average 27% reduction in decayed, missing or filled permanent teeth [22]. Most studies addressing this topic have been conducted in school settings. Due to the widespread use of fluoride-containing toothpastes, supervised rinsing programmes have become less common in most European countries, including Austria. The use of mouth rinsing solutions would require a clear recommendation by dental or pediatric professionals for guardians to follow.

Toothpaste was the primary fluoride source in the study cohort, consistent with findings from other low-fluoride regions [19]. For children over six years, a fluoride concentration of approximately 1400 ppm in toothpaste is recommended, which was used by 76.4% of participants. However, the amount of toothpaste applied was often insufficient. Only 23.1% of participants applied an adequate amount of fluoride via toothpaste, as recommended per the German Society for Preventive Dentistry. No statistically significant difference was found between toothpaste quantity and its fluoride concentration, suggesting that fluoride content was not a determining factor in dosage decisions, likely due to insufficient knowledge The authors advocate for industry measures such as dosage aids and clearer labeling to improve adherence and reduce underdosing, which was 3.46 times more likely with a rice grain-sized amount (< 0.25 g), 1.17 times with a pea-sized amount (0.25–0.5 g), and 0.30 times with the recommended dosage (> 0.5 g). The highest fluoride intake observed (0.4 mg/kg body weight) remained well below the probable toxic dose of 5 mg/kg [23].

A striking lack of awareness regarding the importance of fluoride prophylaxis has been observed among parents and guardians. Only 40.4% correctly identified whether their child’s toothpaste contained fluoride, and merely 43.7% recognized fluoride prophylaxis as necessary. This is in contrast to the actual measures implemented, as only 3.7% did not use any fluoride prophylaxis and brushed with either fluoride-free toothpaste or without toothpaste at all. These findings highlight a clear need for improved parental education regarding fluoride dosage and the potential benefits of additional fluoride sources, such as fluoridated table salt.

The findings of this study are not consistent with those reported in younger age groups. In comparable studies involving children aged 0–6 years, where parents applied toothpaste on behalf of their children, the mean amount dispensed exceeded the recommended quantities—namely, a rice grain–sized smear for infants and a pea-sized amount for preschool-aged children. These findings underscore the pivotal role of fluoride in pediatric oral health and the necessity for a judicious, evidence-based approach to its administration. Rather than indiscriminately advocating for either increased or decreased fluoride exposure in children, emphasis should be placed on enhancing parental knowledge and education regarding age-appropriate fluoride dosing, thereby optimizing therapeutic benefits while minimizing potential risks [24–26].

Limitations of the present study include the reliance on estimated rather than measured dietary fluoride intake, based on average consumption of drinking water and table salt. Additionally, no clinical assessment of caries prevalence or dental fluorosis was performed, facilitating participant accessibility and ensuring a representative sample size. Future studies should correlate daily fluoride exposure with caries incidence in middle-aged children. Given that in 2016 only 55% of six-year-olds in Austria were caries-free [27], it is evident that caries prophylaxis strategies require optimization.

## Conclusion

The present study highlights the urgent need to educate and raise awareness among parents and guardians about the correct caries-preventive use of fluorides. In areas without tap water fluoridation middle-aged children should use at least 1 cm (> 0.5 g) of fluoridated toothpaste (> 1400 ppm) twice daily, supplemented with an additional fluoride source such as fluoridated table salt, mouthwash or fluoride gel to achieve optimal caries prevention outcomes.

## Data Availability

No datasets were generated or analysed during the current study.

## Declarations

### Study approval statement

This study protocol was reviewed and approved by the Ethics committee of the Medical University of Innsbruck, Austria, approval number EK 1417/2021.

## References

[1] Wen PYF, Chen MX, Zhong YJ, Dong QQ, Wong HM (2022) Global burden and inequality of dental caries, 1990 to 2019. J Dent Res 101(4):392–399. 10.1177/00220345211056247 34852668 10.1177/00220345211056247

[2] World Health Organization (2019) Ending childhood dental caries: WHO implementation manual, Geneva. CC BY-NC-SA 3.0IGO, Licence

[3] Walsh T, Worthington HV, Glenny AM, Marinho VC, Jeroncic A (2019) Fluoride toothpastes of different concentrations for preventing dental caries. Cochrane Database Syst Rev 3(3):CD007868. 10.1002/14651858.CD007868.pub3 30829399 10.1002/14651858.CD007868.pub3 PMC6398117

[4] Pollick H (2018) The role of fluoride in the prevention of tooth decay. Pediatr Clin North Am 65(5):923–940. 10.1016/j.pcl.2018.05.014 30213354 10.1016/j.pcl.2018.05.014

[5] Kaiser D, Neumeister L, Stößer L, Hetzer G (2006) Fluoridkonzentration Im Speichel und in der Plaque nach Verzehr Fluoridsalzhaltiger Speisen. Oral Prophyl 28:110–114

[6] Mah VK, Ford-Jones EL (2012) Spotlight on middle childhood: rejuvenating the ‘forgotten years’. Paediatr Child Health 17(2):81–83. 10.1093/pch/17.2.81 23372398 10.1093/pch/17.2.81 PMC3299351

[7] DelGiudice M (2018) Middle Childhood: An Evolutionary-Developmental Synthesis. In: Halfon N, Forrest CB, Lerner RM, Faustman EM (ed) Handbook of Life Course Health Development, Cham (CH), pp. 95–107

[8] Berg B, Cremer M, Flothkötter M, Koletzko B, Krämer N, Krawinkel M, Lawrenz B, Przyrembel H, Schiffner U, Splieth C, Vetter K, Weißenborn A (2021) Kariesprävention Im Säuglings- und frühen kindesalter. Monatsschr Kinderheilkd 169(6):550–558. 10.1007/s00112-021-01167-z

[9] Deutsche Gesellschaft für Präventivzahnmedizin (2024) Fluoridempfehlungen| Kariesprophylaxe mit Fluorid für jedes Alter. https://www.dgpzm.de/news-und-presse/news/fluoridempfehlungen. Accessed 03.03.2025

[10] EFSA Panel on Dietetic Products Nutrition and Allergies (2013) Scientific opinion on dietary reference values for fluoride. EFSA J 11(8). 10.2903/j.efsa.2013.3332

[11] O’Mullane DM, Baez RJ, Jones S, Lennon MA, Petersen PE, Rugg-Gunn AJ, Whelton H, Whitford GM (2016) Fluoride and oral health. Community Dent Health 33(2):69–99. https://www.ncbi.nlm.nih.gov/pubmed/27352462 27352462

[12] Wong MC, Glenny AM, Tsang BW, Lo EC, Worthington HV, Marinho VC (2010) Topical fluoride as a cause of dental fluorosis in children. Cochrane Database Syst Rev 2010(1):CD007693. 10.1002/14651858.CD007693.pub2 20091645 10.1002/14651858.CD007693.pub2 PMC8078481

[13] Do LG, Sawyer A, John Spencer A, Leary S, Kuring JK, Jones AL, Le T, Reece CE, Ha DH (2024) Early childhood exposures to fluorides and cognitive neurodevelopment: A Population-Based longitudinal study. J Dent Res 104(3):243–250. 10.1177/00220345241299352 39692252 10.1177/00220345241299352 PMC11843800

[14] Deutsche Gesellschaft für Ernährung Referenzwert Wasser https://www.dge.de/wissenschaft/referenzwerte/wasser/. Accessed 03.03.2025

[15] World Health Organization (2022) 5 recommendations to reduce salt intake to live longer and healthier lives. https://www.who.int/europe/news/item/14-03-2022-5-recommendations-to-reduce-salt-intake-to-live-longer-and-healthier-lives. Accessed 03.03.2025

[16] World Health Organization (2022) Guidelines for drinking–water quality: fourth edition incorporating the first and second addenda. ed + 1st add + 2nd add, 4 edn. World Health Organization, Geneva 35417116

[17] Murray JJ, World Health Organization, Federation International Dental, Kellogg WK, Foundation (1986) Appropriate use of fluorides for human health / edited by Murray JJ, World Health Organization, Geneva

[18] Murray JJ (1993) Efficacy of preventive agents for dental caries. Systemic fluorides: water fluoridation. Caries Res 27 Suppl 12–8. 10.1159/000261594 10.1159/000261594 8500120

[19] Maguire A, Zohouri FV, Hindmarch PN, Hatts J, Moynihan PJ (2007) Fluoride intake and urinary excretion in 6- to 7-year-old children living in optimally, sub-optimally and non-fluoridated areas. Community Dent Oral Epidemiol 35(6):479–488. 10.1111/j.1600-0528.2006.00366.x 18039290 10.1111/j.1600-0528.2006.00366.x

[20] Steiner M, Menghini G, Marthaler TM (1989) [The caries incidence in schoolchildren in the Canton of glarus 13 years after the introduction of highly fluoridated salt]. Schweiz Monatsschr Zahnmed 99(8):897–901 2799352

[21] Wegehaupt F, Menghini G (2020) [Fluoride update]. Swiss Dent J 130(9):677–683 32893610 10.61872/sdj-2020-09-02

[22] Marinho VC, Chong LY, Worthington HV, Walsh T (2016) Fluoride mouthrinses for preventing dental caries in children and adolescents. Cochrane Database Syst Rev 7(7):CD002284. 10.1002/14651858.CD002284.pub2 27472005 10.1002/14651858.CD002284.pub2 PMC6457869

[23] Whitford GM (1992) Acute and chronic fluoride toxicity. J Dent Res 71(5):1249–1254. 10.1177/00220345920710051901 1607442 10.1177/00220345920710051901

[24] Sudradjat H, Meyer F, Fandrich P, Schulze Zur Wiesche E, Limeback H, Enax J (2024) Doses of fluoride toothpaste for children up to 24 months. BDJ Open 10(1):7. 10.1038/s41405-024-00187-7 38296947 10.1038/s41405-024-00187-7 PMC10831090

[25] Adé DC, Filippi C, Filippi A (2024) A survey on toothbrushing practices and dosing of fluoridated toothpaste among preschool children in the cantons of Basel-Stadt and berne, Switzerland. Swiss Dent J 133(2):18–34. 10.61872/sdj-2024-07-08-01 37909276 10.61872/sdj-2024-07-08-01

[26] Creeth J, Bosma ML, Govier K (2013) How much is a ‘pea-sized amount’? A study of dentifrice dosing by parents in three countries. Int Dent J;63 Suppl 2(Suppl 2):25–30. 10.1111/idj.12074 10.1111/idj.12076 PMC9375012 24283281

[27] Bodenwinkler A, Sax G, Kerschbaum J (2017) Länder Zahnstatuserhebung 2016: Sechsjährige in Österreich. Zahnstatus sechsjähriger Kinder mit und ohne Migrationshintergrund. https://goeg.at/sites/goeg.at/files/2017-11/L%C3%A4nder-Zahnstatuserhebung_FINAL.pdf. Accessed 03.03.2025)

